# Community Health and Employee Work Performance in the American Manufacturing Environment

**DOI:** 10.1007/s10900-018-0570-5

**Published:** 2018-09-07

**Authors:** Megan McHugh, Dustin D. French, Diane Farley, Claude R. Maechling, Dorothy D. Dunlop, Jane L. Holl

**Affiliations:** 1 Center for Healthcare Studies, Northwestern University, Feinberg School of Medicine, Chicago, IL USA; 2 Center for Health Care and Policy Research, Penn State College of Health and Human Development, University Park, PA USA; 3 Branstad Family Foundation, Chicago, IL USA

**Keywords:** Community health, Public health, Case study, Mixed methods, Manufacturing, Absenteeism

## Abstract

Although better community health has long been assumed to be good for local businesses, evidence demonstrating the relationship between community health and employee performance is quite limited. Drawing on human resources data on 6103 employees from four large US manufacturing plants, we found that employees living in counties with poor community health outcomes had considerably higher rates of absenteeism and tardiness (ABT). For example, in one company, employees living in communities with high rates of children on free or reduced lunch had higher rates of ABT compared to other employees [adjusted odds ratio (OR) 2.76, 95% confidence interval (CI) 2.52–3.04], and employees living in communities with high rates of drug overdose deaths had higher rates of ABT (OR 1.51, 95% CI 1.29–1.77). In one plant, the annual value of lost wages due to ABT was over $1.3 million per year. Employees reported that poor community health (e.g., poverty, caregiving burdens, family dysfunction, drug use) resulted in “mental stress” leading to distraction, poor job performance, and more rarely, lapses in safety. These findings bolster the case for greater private sector investment in community health.

## Introduction

For decades, researchers and consultants have produced data showing that poor employee health leads to high direct (e.g., health claims) and indirect (e.g., absenteeism) costs [1, 2]. In response, more than 80% of large companies offer employee wellness benefits, and many offer incentives to employees for participation [3]. However, even when an employer implements health-promoting strategies at the worksite, many employees then go home to unhealthy neighborhoods, and the workplace progress is compromised [4]. As a result, researchers and advocates have made a strong case for business investment in community health [5–9].

Although the link between community health and employee performance (e.g., absenteeism, tardiness, productivity) is conceptually appealing—healthy communities produce healthy workers—evidence demonstrating the effect of community health on employee performance is quite limited [10]. One study found that workers are likely to be in poor health if they live in counties with a poor health profile [11]. A recent dialogue session with 54 executives from 47 organizations revealed that the primary barrier that limits employers from playing a larger role in advancing community health is a lack of understanding of the connection between community health and its impact on business [12]. With better evidence demonstrating the relationship between community health and employee performance, the financial rationale may be clearer for businesses to establish policies, partnerships, and programs focused on community health.

The purpose of our study was to generate this evidence. Using a comparative case study design of four manufacturing plants, our data collection and analyses were aimed at examining whether (1) plants located in counties with relatively poor health outcomes experienced poorer worker performance (e.g., absenteeism, tardiness), and (2) the extent to which poorer employee performance was driven, in part, by the health status of the county.

## Methods

### Case Selection

This comparative case study included two manufacturing companies, which each contributed data from two U.S. plants. We focused on manufacturing because it is a large industry employing over 12 million workers [13], and previous work has shown that manufacturing communities have relatively high rates of poor health behaviors and health outcomes [11]. Additionally, we limited our inclusion criteria to manufacturing plants that serve as an “anchor” within their community, meaning that each plant employs a large share of local residents, and dominates the economic activity of their communities. Communities with anchor companies may be particularly well suited to examine the link between community health and employee performance. First, as the major employer and purchaser of health services in the community, anchor companies may be more directly impacted by changes in health status of a community, for example, through health spending, absenteeism, and productivity. Second, anchor companies typically remain in the same community for decades, which means that they influence the history and culture of the community. Furthermore, anchor company executives typically have close ties to community leaders, who are key partners in any strategy to promote healthy populations and build healthy communities.

Two manufacturing companies that met the above criteria participated in the study. In partnership with company leaders, we reviewed community health data from the Robert Wood Johnson Foundation’s County Health Rankings and Roadmaps (CHRR) for the counties in which their largest plants were located. For each company, we selected one plant located in a community with relatively good community health outcomes, and one plant located in a community with relatively poor community health outcomes. These four non-union plants and communities represented our cases.

### Data Collection

The companies provided employee-level data on age, sex, race, number of years employed by the company, hourly wage, zip code, and 5 years of data on absenteeism and tardiness (ABT) for 6103 hourly employees (i.e., non-management) across the four manufacturing plants. We also obtained county-level health measures from the CHRR, and merged them with the employee-level data using a zip-to-county crosswalk from the U.S. Department of Housing and Urban Development’s Office of Policy Development and Research. The four community health measures were the percent of children on free or reduced lunch (proxy for economic stability), rate of drug overdose deaths (proxy for social and community context), and physical inactivity and adult smoking rates (proxies for health and health behaviors).

We conducted 2 day site visits to each plant and conducted interviews with 12 plant managers and held two focus groups with front-line, hourly employees (4–12 participants per focus group). The interviews and focus groups were guided by semi-structured protocols available upon request. The protocol included questions about the drivers of employee performance, ABT, and the influence of employee health on the workplace. Additionally, all participants were shown local, county-level data from the CHRR, and asked to comment on how community health affects the work at the plant. The interviews lasted, on average, 35 min, and the focus groups, 75 min. The study was approved by Northwestern University’s Institutional Review Board, and informed consent was obtained from all participants in the interviews and focus groups.

### Analytic Approach

The key measure of employee performance was ABT per 1000 hours worked, where a year of full-time work is 2080 hours. ABT is recorded in time keeping systems kept by the human resources division of the companies. In these systems, ABT was defined as hours when the employee failed to report for scheduled work and failed to follow the company policy of notifying the team manager or designee. ABT does not include vacation, sick time, or short- or long-term disability days. We used a nested fixed effects model to estimate the relationship between each community health measure and ABT per 1000 hours worked. The analysis was limited to employees with at least 12 months of service to the company. To facilitate analysis and interpretation of results, the community health measures were converted to binary measures, indicating whether the employee resided in an outlier county with a value that exceeded the state average. The model also controlled for employee characteristics, including categorical variables for age and years at the company, and binary variables for gender and race (black vs. non-black). Estimates on lost wages due to ABT were based on recorded ABT hours from human resources data multiplied by the employee hourly wage. Analyses were conducted using SAS®, version 9.4 Cary, NC.

All interviews and focus groups were digitally recorded and uploaded to Atlas.ti, a qualitative software program. We used thematic analysis, a systematic search for themes, patterns, and repetitions in the data [14]. We used a combination of deductive codes, based on the interview protocol, and inductive codes based on new concepts that emerged following an initial reading of the transcripts [15]. The two team members who conducted the site visits (MM, DF) applied the codes to two interview transcripts, and their work was compared to ensure a common understanding of the coding definitions. A single coder continued to apply the codes to the remaining interview transcripts [15]. All focus group transcripts were double-coded by the team members, and discrepancies were resolved through discussion.

## Results

A profile of the hourly workers with at least 12 months of service is shown in Table 1. Although there was variation in the number of employees at each plant, all four plants were large and overwhelmingly employed men. The average age of employees ranged from 38.2 to 46.3 years, and average number of years with the company ranged from 6.0 to 10.8 years. There were large differences in the percent of workers who were black, ranging from 4.2 to 84.8%. Employees at plants A and C lived in counties that had poorer community health indicators, compared to their company peers in plants B and D.

**Table 1:** Profile of plant employees and community health

	Company 1		Company 2	
	Plant A	Plant B	Plant C	Plant D
Employee profile				
Number of hourly employees	2401	1082	979	1641
Percent male	83%	81%	62%	76%*
Percent black	5.5%	27.9%*	84.8%	4.2%*
Mean age	38.2	45.8*	46.3	46.2
Median employee years with the company	6.0	10.8*	9.7	8.8*
Weighted average of employees’ county-level community health measures				
Percent of children on free or reduced lunch	67.6	50.1%*	77.9%	52.4%*
Drug overdose deaths per 10,000	30.3	20.2*	15.6	13.6*
Physical inactivity	31.2%	24.9%*	21.1	17.6*
Adult smoking	19.0%	16.8%*	30.6	25.2*

*Indicates statistically significant difference (p < .05) from the other plant within the same company

### Differences in Organizational Stress (i.e., ABT) by Plant Location

Within each company, the average rate of ABT was higher at the plant located in the community with relatively poor health outcomes (Table 2). However, rates of ABT were generally low, with the exception of plant A, which had an average of 15.5 hours of ABT per 1000 hours worked. These findings were supported by the interviews with managers. Managers from plant A frequently described employee absence as a problem, while managers from the other plants did not. The annual value of the lost wages ranged from $3489 in plant D to over $1.3 million in plant A.

**Table 2:** Absenteeism and tardiness in the four plants

	Company 1		Company 2	
	Plant A (N = 2401)	Plant B (N = 1082)	Plant C (N = 979)	Plant D (N = 1641)
Average hours of ABT per 1000 hours worked	15.5	0.8*	0.3	0.2*
Annual dollar value of lost wages	$1,334,534	$31,607	$5897	$3489
Manager experiences	“We have a real attendance problem. I have three machines that I run daily, sometimes I have enough people show up where I only can run two.” “In my previous business unit, the largest factor for my inability to function, was people just not coming to work.”	“[We give employees] a verbal, then a written warning, a final, and then out the door. I’ve never had to go past written on anybody.”	“In my department, I would say [attendance] is not an issue. That doesn’t mean that we don’t have certain individuals who are not meeting attendance performance expectations; however, as an overall stopping the business because of poor attendance, no it’s not the problem.”	“Not showing up for work …can lead to many things as far as disciplinary action. Now I can say in all my years I’ve only had one instance where the employee did not call in and it had—it was just a family tragedy thing that happened.”

*Indicates statistically significant difference (p < .05) from the other plant within the same company

### Factors Associated with Organizational Stress

The youngest employees, relative to the oldest, had 51% greater ABT in company 1 and 66% greater ABT in company 2 (Table 3). Similarly, employees with the fewest years of service to the company, compared to those with the most years of service, had more than two times the amount of ABT at company 1, and more than three times the amount of ABT at company 2. ABT for males was less than for females in both companies, and blacks had 32% more ABT in company 2, but less ABT in company 1.

**Table 3:** Company and plant nested multivariate results on the rate of absenteeism or tardiness (ABT) per 1000 hours worked

	Company 1	Company 2
	ABT estimate (95% confidence interval)	ABT estimate (95% confidence interval)
Employee characteristics		
Employee age		
Quartile 1 (youngest)	1.51 (1.30–1.74)	1.66 (1.45–1.91)
Quartile 2	1.39 (1.22–1.59)	1.60 (1.45–1.76)
Quartile 3	1.24 (1.10–1.40)	1.35 (1.24–1.46)
Quartile 4 (oldest)	Ref.	Ref.
Employee years with company		
Quartile 1 (fewest years)	2.24 (1.94–2.59)	3.38 (3.01–3.79)
Quartile 2	1.43 (1.24–1.64)	1.58 (1.42–1.76)
Quartile 3	1.26 (1.11–1.42)	1.06 (0.97–1.16)
Quartile 4 (most years)	Ref.	Ref.
Male	0.85 (0.77–0.94)	0.93 (0.86–0.99)
Black	0.65 (0.58–0.73)	1.32 (1.17–1.48)
County-level community health measures		
Children on free or reduced lunch exceeds state median	2.76 (2.52–3.04)	1.69 (1.39–2.04)
Drug overdose death rate exceeds state median	1.51 (1.29–1.77)	1.52 (1.15–2.00)
Physical inactivity rate exceeds state median	2.61 (2.38–2.87)	1.00 (0.90–1.11)
Adult smoking rate exceeds state median	1.47 (1.27–1.70)	0.83 (0.70–1.0)

For company 1, employees living in counties where the percent of children receiving free or reduced lunches exceeded the state median had 276 percent higher ABT than employees who lived in counties where the percent of children receiving free or reduced lunches did not exceed the state median. In company 2, employees living in counties where the percent of children receiving free or reduced lunches exceeded the state median had 69 percent more ABT. Employees residing in counties where the rate of drug overdose deaths exceeded the state median had 51% and 52% more ABT in companies 1 and 2, respectively. Results for physical inactivity and smoking were mixed. At company 1, both measures were associated with higher ABT, whereas at company 2 they were indeterminate.

When we asked managers and front-line employees about the drivers of unexcused or unplanned absences, most respondents from all four plants pointed to deficiencies in the work ethic among many younger workers. According to one manager:

I hate to say, the kids today aren’t what they used to be, but the maturity of the group is less than I’ve ever seen. I’ve been here for 19 years. Nine of it has been close to some form of management. Just in the nine years, I can’t believe the maturity level of these people. They don’t have the same drive.

Several respondents noted that many younger workers still live with their parents, and the need for a steady paycheck was not as strong, and the consequences for missing work were not as compelling. Many respondents speculated that lapses in work ethic, and resulting unexcused absences, were driven by the environment in which younger workers were raised:

It’s not like it used to be, where they grew up doing farm chores and different things like that. They’re coming in. They’re very—sedentary...Now all the sudden, it’s like the reality of a physical job that they’ve never done before is—it’s a shock to their system.

There were two additional drivers of unexcused absences frequently reported by respondents from plant A. The first pertained to worker stress associated with family issues, including divorce and drug use. The second pertained to company policies, both leniency regarding unexcused absences and reductions in pay and benefits, which led employees to devalue attendance and employment (Table 4).

**Table 4:** Additional drivers of absenteeism and tardiness (ABT) in company 1, plant A

Stress Associated with Family Issues
“Actually, that’s probably our number one issue with people now missing work, because of stress-related issues, maybe from an outside family issue. I mean, I hate to say this, but more people are getting into drugs and alcohol abuse, and stuff like that. That’s probably been the majority, as far as people that has missed recently has been stuff like that….I mean, there was a lady who quit the other day, because of her family, a family member that, you know, today she felt like she needed to stay home and take care of her family member, because they have a bad drug problem. She’s been off for 3 months, because of stress-related, because of her family member.”
“Last night a lady called me and her 15 year-old son had been arrested, so, she need to go pick him up out of jail so she wasn’t going to make it to work. Have one last week, a young man caught his wife cheating. A perfect example, that particular case, his attendance had been trending in a bad direction, you see what happened.”
“Like I said, the stress. I think the mental stress of outside factors. That to me is the main thing that I have seen, like I said, the last couple of years of people taking off from mental stress.”
Company policies
“There are departments where disciplinary action never comes about.”
“Before we do any type of discipline for them, we give them every opportunity to try and make it good, and then you’ll have people who just aren’t able to do so. I had one employee who was absent 18 different times with different issues. I was very accommodating to try and make sure everybody has what they need and are able to come to work, but at the same time, that’s also a downfall, too, with people not respecting those boundaries.”
“When we were hired, [name of company] was the place to work.” “I waited 5 years before I got hired.” “You didn’t rotate [shifts].” “The benefits were some of the best in the area. The pay was the best in the area. The vacation, all these things were. Well, now it’s kinda like well, we’re supposed to be like everybody else. Down here with everybody else. We’re comparable.”
“[Name of company] cut the pay of the people, and the quality of people that we started getting was not what it used to be.”

When we asked respondents from all four plants about how the health of the community affects the work within the plant, we frequently heard about “mental stress.” Respondents described stresses associated with caring for young children or family members in poor health, the burden of a family member with a drug or alcohol addiction or who is incarcerated, divorce and domestic violence issues, and financial problems. Respondents noted that these external stressors (often combined with job-related stress) caused workers to be distracted, which many respondents said contributed to poor job performance and occasional lapses in safety.

## Discussion

In an attempt to elucidate the relationship between community health and employee performance, we conducted a comparative case study of four manufacturing plants and communities. We found that plants located in communities with relatively poor community health profiles had higher rates of ABT. Our quantitative and qualitative findings lend support to the notion that employee performance is affected by community health.

Although we can demonstrate a link between community health and employee performance, the dollar value of lost wages associated with ABT was relatively low for three of the four plants included in this study. However, our cost estimates are likely understated. If a worker is unexpectedly absent, the company may “call in” another worker, paying time-and-a-half or double time to cover the shift. Further, in plant A we heard about absenteeism leading to a direct reduction in the production of goods. Our cost estimates do not take into account lost sales or the cost of labor variances.

The opportunity to address deficiencies in community health in manufacturing communities is substantial. Prior work suggests that manufacturing communities have higher rates of poor health behaviors, such as smoking and physical inactivity, and health outcomes, such as diabetes and cardiovascular deaths [11]. Our findings also point to high levels of stress experienced by hourly workers due to family and community issues, which may be underlying the poor health behaviors and health outcomes. Within the plants, this stress is manifested through absences, a lack of focus, low morale, and in rare cases, accidents. This is consistent with research showing that chronic stress is associated with fatigue, an inability to concentrate, and irritability [16–18].

Our results may be used to bolster the case for greater business investment in community health, particularly in communities like plant A’s where the connection was most identifiable. Large employers, including manufacturers, may rightly maintain that they are already directing significant sums of corporate philanthropy toward improving the social determinants of health [19–21]. However, their investments in local community health are typically made without the benefit of rigorous evidence or evaluation [19, 20]. Further, the social problems affecting organizational stress, including poverty and drug use, are complex, intractable problems that are unlikely to be mitigated solely through companies acting on their own.

Nevertheless, there is an opportunity for greater business leadership in addressing the social determinants of health, in addition to simply increasing corporate philanthropy. First, employers can work more closely with public health partners, which have extensive experience monitoring the health status of communities, developing policies and partnerships to address community health, and evaluating the effectiveness of population-based interventions [22]. Second, large employers can use their considerable collective political might to advocate for greater investment in public health [23, 24]. Despite evidence on the cost-effectiveness of public health spending [25–27], public health has been chronically underfunded, representing a small portion of federal health spending [28, 29]. Third, employers should also reconsider their wellness benefits, especially since previous studies have shown that they rarely yield the desired results [30, 31]. Wellness benefits could be enhanced and customized locally to include well-being components that aim to mitigate the outside stressors on employees and their families.

Employers will need assistance to make this shift a reality. First and foremost, more research is needed to identify the most effective interventions for improving community health. Our findings suggest that interventions targeting youth may be attractive to employers, as younger workers had higher rates of ABT, and were often cited as having a poor work ethic.

This study had several limitations. First, our single measure of employee performance, ABT per 1000 hours worked, was selected because similar data were available across all four plants. There may be other indicators that better represent employee performance, for example, disciplinary action, complaints received, and short- and long-term disability. Second, the timekeeping systems and disability policies differed between the two companies, making it difficult to make direct comparisons of ABT across companies. Third, the case study was designed as a first effort to test two hypotheses regarding community health and employee performance within two companies. Replication is needed to test the generalizability of the findings. Finally, the observed statistical relationships between community health and ABT were associational, and do not necessarily indicate causal mechanisms.

## Conclusion

Across four large manufacturing plants, we found that employee performance was affected by community health. Employees living in counties with high rates of poor community health outcomes had considerably higher rates of ABT. Employees reported that poor community health (poverty, caregiving burdens, family dysfunction, drug use) resulted in “mental stress” leading to distraction, poor job performance, and more rarely, lapses in safety. Our findings bolster the case for greater private sector investment in community health. However, additional research is needed to identify the most effective interventions that businesses can undertake to improve community health.
